# Activated Clotting Time (ACT) for Monitoring of Low-Dose Heparin: Performance Characteristics in Healthy Adults and Critically Ill Patients

**DOI:** 10.1177/1076029620975494

**Published:** 2020-12-22

**Authors:** Johannes E. Wehner, Martin Boehne, Sascha David, Korbinian Brand, Andreas Tiede, Rolf Bikker

**Affiliations:** 1 Department of Hematology, Hemostasis, Oncology and Stem Cell Transplantation, Hannover Medical School, Hannover, Germany; 2 Department of Pediatric Cardiology and Intensive Care Medicine, Hannover Medical School, Hannover, Germany; 3 Department of Nephrology and Hypertension, Hannover Medical School, Hannover, Germany; 4 Institute of Clinical Chemistry, Hannover Medical School, Hannover, Germany

**Keywords:** activated clotting time, anticoagulants, blood coagulation, point-of care systems, blood coagulations tests, heparin

## Abstract

Dose adjustment of unfractionated heparin (UFH) anticoagulation is an important factor to reduce hemorrhagic events. High doses of heparin can be monitored by Activated Clotting Time (ACT). Because of limited information about the monitoring of low-dose heparin we assessed monitoring by ACT, aPTT and anti-Xa. Blood samples from healthy volunteers (n = 54) were treated ex vivo with increasing UFH doses (0-0.4 IU/ml). Samples from ICU-patients (n = 60), were drawn during continuous UFH infusion. Simultaneous ACT measurements were performed using iSTAT and Hemochron. In UFH treated blood, iSTAT and Hemochron showed a significant change of ACT at ≥0.075 IU/ml and ≥0.1 IU/ml UFH, respectively. In ICU-patients no relationship between ACT and either UFH dose, aPTT and anti-Xa was observed. Hemochron was affected by antithrombin and platelet count. iSTAT was sensitive to CRP and hematocrit. A moderate correlation was identified between UFH dose and aPTT (R^2^ = 0.196) or anti-Xa (R^2^ = 0.162). In heparin-spiked blood, ACT is sensitive to heparin at levels of ≥0.1 IU/ml heparin. In ICU-patients, ACT did not correlate with UFH dose or other established methods. Both systems were differently influenced by certain parameters.

## Introduction

Extracorporeal life support systems are increasingly used in modern intensive care medicine both in the “bridge to recovery” and “bridge to transplant” context.^1^ Implementing an extracorporeal circuit in critically ill patients - that regularly show diverse coagulopathies as part of their disease - further exacerbates hemostatic imbalances due to the interaction between blood and artificial surfaces.^2^ To prevent thromboembolic processes infusion of unfractionated heparin (UFH) at therapeutic dosages (e.g. 20-50 IU/kg/h) is recommended.^3^ Given that any heparin administration increases the general risk of bleeding complications^4^ it is obvious that many centers prefer low-dose heparin or even an anticoagulant-free management in order to reduce hemorrhagic events.^4–7^

Classically, low-dose and subtherapeutic UFH anticoagulation is not monitored at all.^8^ Previous studies, focusing on adjusted low-dose UFH prophylaxis, suggested that UFH monitoring may improve the outcome in critical ill patients.^9,10^ Typically, UFH is monitored using anti-Xa and aPTT. However, these methods regularly performed in the central laboratory claim time and are often out of the quantification limit. Here, especially point-of-care activated clotting time (ACT) provides a potential benefit due to its decentralized and time-efficient coagulation analysis.

Monitoring of low-dose UFH by point-of-care ACT is not established and there is only limited information about monitoring and interpretation of corresponding coagulations markers. Currently, there are various analytical ACT devices available and each system uses different methods to detect clot formation. In this context, it was already demonstrated that specific devices are not comparable in the therapeutic high-dose UFH context.^11^ However, the suitability of ACT in the low-dose heparin range has not been investigated yet.

In a cohort of healthy individuals and ICU-patients, we exploratory investigated the use of standard laboratory coagulation markers (anti-Xa, aPTT) and 2 modern point-of-care ACT monitoring-systems in low-dose UFH anticoagulation.

## Methods and Materials

### Study Design

This explorative observational study was performed at Hannover Medical School and approved by the university ethics committee (study no. 8449_BO_S2019). Informed and written consent was obtained from healthy volunteers and patients of appropriate age or, in case of minors or high morbidity patients, by official representatives. Information included study objectives and protocol, risk-benefit ratio, voluntary participation and privacy policy. For ex vivo heparin anticoagulation, healthy individuals (heparin-spiked blood population) ranging from 18 to 75 years provided venous blood samples. The presence of acute illness, known coagulopathy or medication with anticoagulants and platelet aggregating drugs were exclusion criteria. For the in vivo part, blood was collected from critical ill patients with constant heparin anticoagulation treatment (> 4 hours). A requirement for trial participation was the necessity of daily heparin dose monitoring. ICU-patients with known congenital or acquired coagulation disorders potentially affecting ACT, aPTT or anti-Xa results were excluded. Thrombocytopenia however, was not an exclusion criterion.

### Blood Sampling

For ex vivo evaluation, 4 ml of venous whole blood was collected via a peripheral punction. Following collection, the sample was immediately transferred and gently mixed in a plastic tube with no coagulation-activating surfaces containing prefilled UFH (Heparin-sodium-5000-ratiopharm^®^) to obtain final heparin concentrations of 0-0.4 IU/ml. Here, one volunteer of the heparin-spiked population provide blood-samples for different heparin levels. Mixed blood sample was directly transfered to the prepared ACT analyzers and immediately collected in a citrate tube for laboratory measurements. Citrate tubes were centrifuged and analyzed with the appropriate method within the central laboratory. Samples without heparin were obtained directly into the citrate tube or a syringe to fill the ACT cartridges.

Measurements in critically ill patients were conducted with blood samples directly collected from arterial or central venous catheters. In order to avoid contamination with heparin or irrigation fluid, the sample was never taken from a heparin branch of the central venous access and the first 4 ml of blood were discarded. ACT measurements were performed during clinical routine diagnostic, including a citrate tube for coagulation analysis. To measure ACT whole blood was separately collected in a syringe an applied on the ACT measurement device. Specifically to reduce volume loss in pediatric intensive care patients, residual whole blood from routine diagnostic collected in a syringe was applied on the ACT devices and immediately transferred to a citrate tube.

### Determination of Activated Clotting Time

For ACT measurements iSTAT Alinity (Abbott Point of Care, Princeton, NJ) and Hemochron Signature Elite (Accriva, San Diego, CA) were used. Immediately after sample collection the ACT was monitored in parallel on at least 2 prepared analyzers in a random order (duplicates or triplicates). The iSTAT ACT-cartridge activates a predefined amount of whole blood (40 μl) via the intrinsic activator kaolin. The measuring end point is the ampherometric determination of an electroactive thrombin splitting product as a positive result of clot formation at a temperature of 37°C. With the Hemochron Signature Elite, ACT low range cartridges (Celite activator) for lower heparin dosage ranges were used. Here the sample is applied to the cartridge and automatically transferred to a testing channel where the drop of flow velocity is measured at 37°C.

### Laboratory Analysis

Samples were collected for each measurement using 3.2% citrate tubes (S-Monovette^®^ 2.9 ml 9NC). For aPTT measurements the Pathromtin^®^ SL Assay and Dade^®^ Actin^®^ FS assay, which is less sensitive to lupus anticoagulants, were performed. For anti-Xa measurement the INNOVANCE^®^ Heparin Assay without supplemented antithrombin was applied. Both parameters were measured using the Atellica COAG 360 System (Siemens Healthcare Diagnostics Products, Marburg, GER). For the in vivo evaluation, the endogenous antithrombin activity (INNOVANCE^®^ Antithrombin), daily platelet count, hematocrit and CRP (Tina-quant C-Reactive Protein IV; Roche, Basel, CH) were determined.

### Statistical Analysis

Statistical analyses were performed using SPSS 25.0 (IBM, Armonk, NY, USA) and GraphPad software 5.0 (GraphPad Prism, La Jolla, CA, USA). The sensitivity of mean ACT was interpreted in the ex vivo evaluation using a one-way ANOVA analysis (Tukey’s multiple comparison test). Study results were analyzed by linear regression, where the Standard deviation of the residuals (Sy.x) was determined. To quantify the bias between point-of-care ACT systems, a Bland-Altman plot was performed using % Difference (100*(A-B)/average). Normal distribution was verified by a Kolmogorov-Smirnow test. A p-value ≤ 0.05 was interpreted as significant. Influence of determined parameters on ACT was calculated using multivariate analysis.

## Results

### Study Populations

The blood samples were collected from 54 healthy individuals for ex vivo analysis. Sixty ICU-patients were included to evaluate the monitoring of low-dose anticoagulation strategies in vivo. The clinical measurements were performed both in adult and pediatric critically ill patients in order to analyze a broad spectrum of patients, where low-dose UFH anticoagulation is used to prevent thromboembolic and bleeding events. Therefore, 30% of ICU-patients were < 12 years (89% ≤ 1 year). Patient demographics and baseline characteristics are shown in Table 1.

**Table 1.** Demographics and Baseline Characteristics of Study Population.

Characteristics	Healthy individuals	Adolescents and adult ICU^1^-patients [age ≥ 12 years]	Pediatric ICU-patients [age < 12 years]	All patients
Subjects, n	54	42	18	60
ACT^2^ measurements, n	344	184	73	257
Age, y. median, IQR^3^, (range)	23, 8, (19-61)	45, 31, (13-77)	0, 1, (0-9)	32, 55, (0-77)
Male sex, (%)	37	60	61	60
BMI^4^, kg/m^2^ median, IQR, (range)	22.3, 3.2, (18.6-35.1)	25.1, 5.5, (19.1-49.9)	13.5, 3.6, (10.3-24.4)	24.7, 11.8, (10.3-49.9)
**Diagnosis**, n (%)				
Respiratory failure		17 (40)		17 (28)
Congenital heart defect		2 (5)	11 (61)	13 (22)
Sepsis		7 (17)		7 (12)
Cardiac arrest		6 (14)		6 (10)
Heart failure		2 (5)	4 (22)	6 (10)
Thromboembolism		5 (12)		5 (8)
Others		3 (7)	3 (17)	6 (10)
**Heparin indication**, n (%)				
Acute thromboembolism		5 (12)	2 (11)	7 (12)
Device		15 (36)	2 (11)	17 (28)
Atrial fibrillation		4 (9)		4 (7)
Thromboprophylaxis only, and none of the above		16 (38)	11 (61)	27 (45)
More than one of the above		2 (5)	3 (17)	5 (8)
**Heparin dose**, IU/kg/d median, IQR, (range)		218, 266, (96-669)	209, 227, (27-603)	216, 239, (27-669)
**Platelet count**, platelet/µl median, IQR, (range)		144, 140, (15-410)	207, 165, (68-483)	155, 140, (15-483)
**Hematocrit**, % median, IQR, (range)		26.2, 6.2, (18.4-45)	36.9, 9 (23.3-45.6)	28, 12, (18.4-45.6)
**Antithrombin, %** median, IQR, (range)		92.9, 44.6 (20.5-150)	88.6, 25.1, (30.2-116)	91.4, 31.7, (20.5-150)
**CRP** ^8^, mg/l median, IQR, (range)		106, 134, (8-425)	27, 49, (6-128)	69, 108, (6-425)
**Fibrinogen**, g/l median, IQR, (range)		3.65, 1.97, (0.57-9)	2.91, 2.66, (1.2-5.71)	3.16, 2.37, (0.57-9)

^1^ ICU, intensive care unit; ^2^ ACT, activated clotting time; ^3^ IQR, interquartile range; ^4^ BMI, body mass index; ^5^ CRP, c-reactive protein.

Critical ill patients received UFH if at least one of the following indications was present: thromboprophylaxis, acute thromboembolism, atrial fibrillation and device support with extracorporeal membrane oxygenation (ECMO) or mechanical heart valve replacement. Patients undergoing ECMO-therapy supported by veno-venous (n = 9) or veno-arterial (n = 6) mode. The following oxygenator-systems for inpatient ECLS-therapy were used: Rotaflow PLS-System® and Cardiohelp HLS-System® (Maquet Getinge group, Rastatt, GER).

### Heparin-Spiked Blood Population

**Figure 1.**
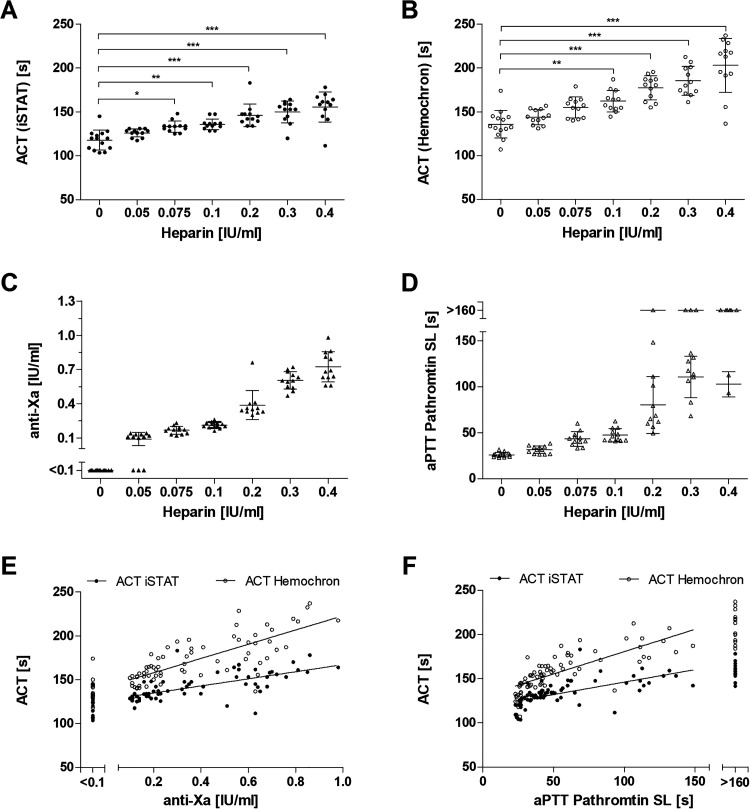
Heparin monitoring via ACT, aPTT and anti-Xa for ex vivo heparin administration. Blood samples were collected from healthy donors (n = 54) and treated with heparin to obtain the indicated heparin concentrations. Two parallel ACT measurements were performed using the iSTAT Alinity (A) and the Hemochron Signature Elite (B) simultaneously. Dots indicate the mean of duplicates, while bars indicate the mean ± standard deviation. Asterisks indicate statistical one-way ANOVA analysis: (*) p ≤ 0.05, (**) p ≤ 0.001, (***) p ≤ 0.0001. Anti-Xa (C) and aPTT (D) measurements were performed on COAG 360 in the central laboratory. The correlation between Hemochron ACT (R^2^ = 0.597) and iSTAT ACT (R^2^ = 0.452) to anti-Xa (E) or Hemochron ACT (R^2^ = 0.596) and iSTAT ACT (R^2^ = 0.412) to aPTT (F) is shown by a linear regression where anti-Xa ≤ 0.1 IU/ml and aPTT ≥ 160 s are excluded.

Significant differences between heparin-free and anticoagulated samples were observed starting at an UFH level of 0.075 IU/ml for the iSTAT Alinity (Figure 1A) and 0.1 IU/ml for the Hemochron (Figure 1B). With increasing heparin concentrations, the differences became more significant on both ACT analyzers (p < 0.05). Concerning the relationship between ACT and UFH dose, we identified a better concordance between UFH concentration and the Hemochron (R^2^ = 0.63) compared to the iSTAT Alinity (R^2^ = 0.542) by linear regression analysis. The inter-individual variability between 2 devices is higher for the Hemochron (SD from 8.23 to 29.43, Sy.x = 16.98) than for the iSTAT Alinity (SD from 4.45 to 16.45, Sy.x = 11.32). Anti-Xa (Figure 1C; R^2^ = 0.8821) and aPTT (Figure 1D; R^2^ = 0.77) measurements demonstrated a higher concordance with UFH concentrations.

Inter-assay comparability evaluation is shown in Figure 1E and F. Here, linear regression analysis for the Hemochron system demonstrated a slightly better concordance to anti-Xa measurements and aPTT results (Table 2). However, considering the 95%-CI and standard deviation of the residuals, the iSTAT Alinity showed a better precision.

**Table 2.** Results of Regression Analysis for Ex Vivo Heparin Administration.

	iSTAT	Hemochron
**anti-Xa**		
Regression Parameters		
Slope	40.47 ± 5.48	81.39 ± 8.24
95% CI^1^	29.51 to 51.43	64.93 to 97.86
Intercept	126.8 ± 2.47	141.7 ± 3.71
95% CI	121.8 to 131.7	134.3 to 149.1
Goodness of fit		
R^2,2^	0.452	0.597
Sx.y^3^	10.90	16.37
**aPTT**		
Regression Parameters		
Slope	0.28 ± 0.04	0.51 ± 0.05
95% CI	0.20 to 0.36	0.41 to 0.61
Intercept	117.9 ± 2.56	130.0 ± 3.16
95% CI	112.8 to 123.0	123.7 to 136.3
Goodness of fit		
R^2^	0.412	0.596
Sx.y	11.03	13.63

^1^CI, confidence interval. ^2^R^2^, statistical parameter to describe the goodness of fit of a regression model.

^3^ Sx.y, standard deviation of the residuals.

**Figure 2.**
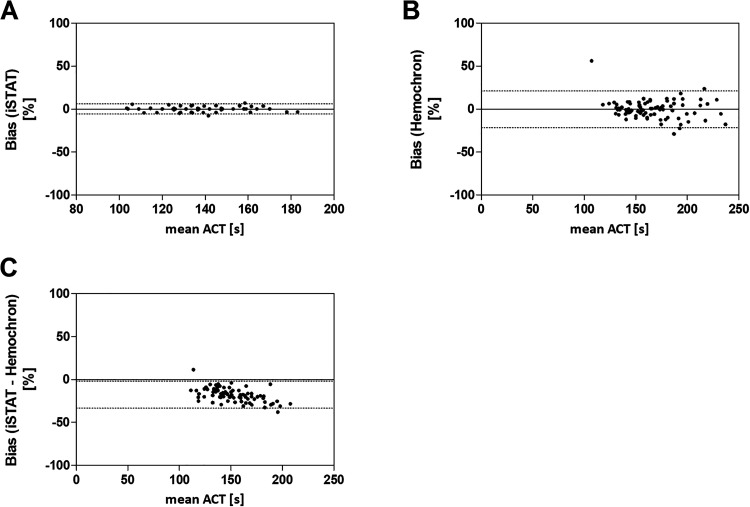
Bland-Altman method comparison between 2 ACT devices (healthy individuals). Blood samples from healthy donors (n = 54) were treated with heparin and 2 parallel ACT measurements were performed simultaneously on each measurement device. The percental bias was analyzed using Bland-Altman plot for iSTAT Alinity (A) with a bias of −0.1423 (lower/upper limit: −5.620 to 5.905), Hemochron Signature Elite with a bias of −0.2213 (lower/upper limit: −21.58 to 21.14) (B) and both devices with a bias of −17.83 (lower/upper limit: −33.57 to −2.077) (C). Dotted lines indicate upper and lower limits.

Evaluating the precision of both ACT devices, we observed a bias of 0.14 ± 2.94% using the iSTAT system (Figure 2A). In contrast, the Hemochron system showed a higher bias of 0.22% with a massively increased SD of 10.9% (Figure 2B), supporting the previous results. Finally, the ex vivo iSTAT measurements were 17.83% lower than parallel performed Hemochron results, with a tendency to increase with higher UFH concentrations (Figure 2C).

### ICU-Patients

**Figure 3.**
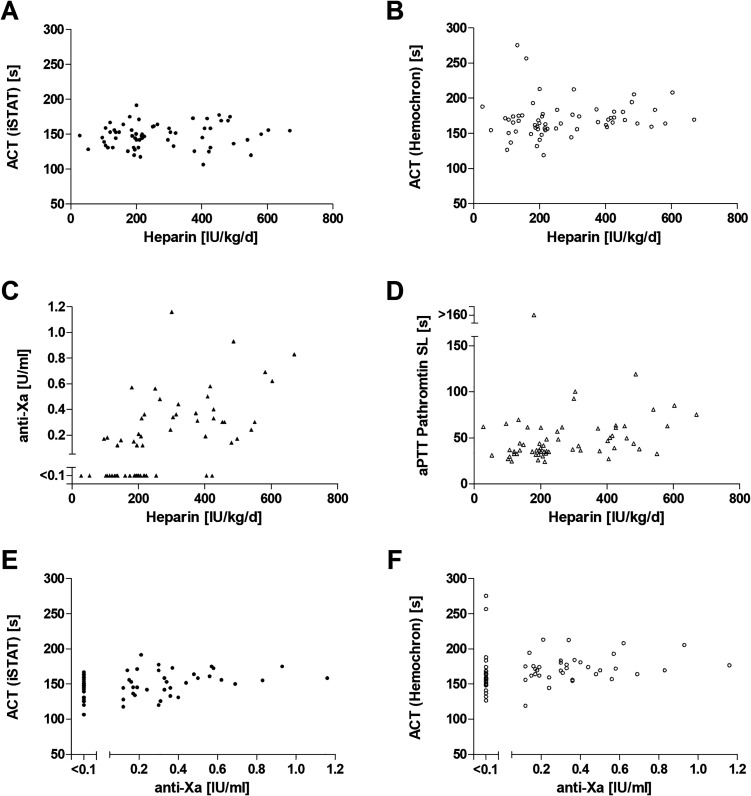
Heparin monitoring via ACT, aPTT and anti-Xa for in vivo heparin administration. Blood samples were collected from ICU-patients (n = 60) treated with low-dose heparin. ACT measurements (mean of n ≥ 2) were performed simultaneously using the iSTAT Alinity (A) and the Hemochron Signature Elite (B). Immediate anti-Xa (C) and aPTT (D) measurement was performed on COAG 360 in the central laboratory and correlated to the iSTAT ACT (E) and the Hemochron ACT (F).

Using the same methods, samples from ICU-patients receiving UFH were analyzed. Here, point-of-care ACT-assays demonstrated no measurable sensitivity for low UFH doses (Figure 3A and B). In contrast, a weak but notable concordance existed between UFH dose and the standard laboratory monitoring (Figure 3C and D). A performed Dave^®^ Actin^®^ FS assay, which is less sensitive to lupus anticoagulant, showed comparable results (Data not shown). However, in the presence of lower heparin doses (< 250 IU/kg/d) anti-Xa results were frequently outside the limit of quantification (< 0.1 IU/ml). Table 3 is summarizing the results of linear regression analysis between UFH dose and all tested monitoring parameters. Comparable to ex vivo analysis, Hemochron demonstrated a higher inter-individual variability. For in vivo analysis, we also tested the inter-assay comparability between anti-Xa and ACT (Figure 3E and F). Significant results were not observed for both point-of-care systems.

**Table 3.** Results of Regression Analysis for In Vivo Heparin Administration.

	iSTAT	Hemochron	anti-Xa	aPTT
Regression parameters				
Slope	0.011 ± 0.015	0.021 ± 0.022	0.0006 ± 0.0002	0.057 ± 0.015
95% CI^1^	−0.018 to 0.041	−0.022 to 0.065	0.0001 to 0.0012	0.026 to 0.087
Intercept	145.1 ± 4.597	164.4 ± 6.865	0.154 ± 0.093	31.78 ± 4.768
95% CI	135.9 to 154.3	150.6 to 178.1	−0.036 to 0.3443	22.24 to 41.33
Goodness of fit				
R^2,2^	0.010	0.016	0.162	0.196
Sx.y^3^	17.26	25.78	0.23	17.75
P-value	0.4422	0.3342	0.0148	0.0004

^1^ CI, confidence interval. ^2^ R^2^, statistical parameter to describe the goodness of fit of a regression model.

^3^ Sx.y, standard deviation of the residuals.

**Figure 4.**
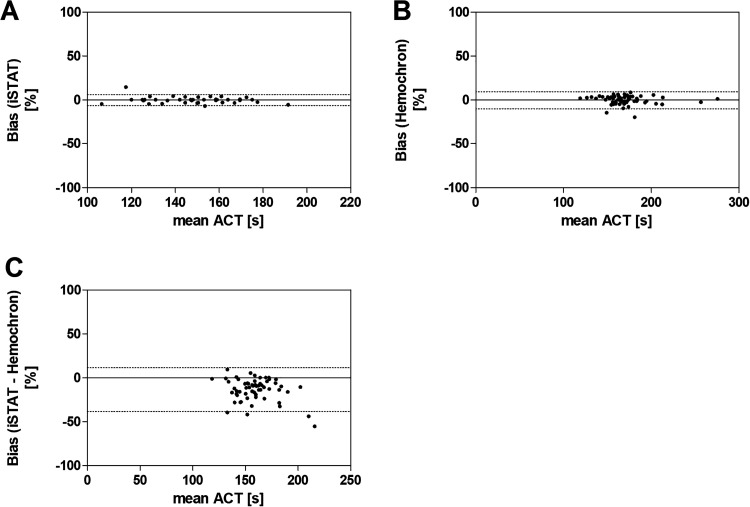
Bland-Altman method comparison between 2 ACT devices (ICU-patients). Blood samples from ICU-patients (n = 60) undergoing low-dose heparin anticoagulation were simultaneously measured with 2 Hemochron signature elite and 2 iSTAT Alinity devices. In the clinical setting the percental bias was analyzed using Bland-Altman plot for iSTAT Alinity (A) with a bias of −0.19 (lower/upper limit: −6.433 to 6.059), Hemochron Signature Elite with a bias of −0.38 (lower/upper limit: −10.15 to 9.380) (B) and both devices with a bias of −13.42 (lower/upper limit: −38.18 to 11.34) (C). Dotted lines indicate upper and lower limits.

Evaluating the precision of both ACT devices in ICU-patients we observed a bias of −0.19 ± 3.82% using the iSTAT Alinity (Figure 4A). Comparable to ex vivo analysis the Hemochron system shows a higher bias of −0.38% with a SD of 4.98% (Figure 4B). Furthermore these measurements were 13.42% higher than parallel performed iSTAT results, with the same tendency as observed in ex vivo analysis (Figure 4C).

To investigate the influence of different factors (heparin-dose, antithrombin, platelet, hematocrit, CRP) on the ACT systems, a multivariate analysis was performed (Table 4). The analysis confirmed that both systems were influenced by UFH dose. Interestingly, a relative importance of the factors hematocrit and CRP was found for iSTAT, whereas Hemochron showed a relative importance on antithrombin and platelet count.

**Table 4.** Suggested Factors Influencing ACT Measurement Device Determined by Multivariate Analysis.

	iSTAT		Hemochron	
Goodness of fit				
R^2, 1^	0.28		0.39	
Relative Importance^2^				
	Rel. importance	P-value	Rel. importance	P-value
Heparin dose	0.12	0.099	0.18	0.013
Antithrombin	0.16	0.064	0.63	<0.001
Platelet count	0.01	0.577	0.17	0.015
Hematocrit	0.40	0.004	0.02	0.023
CRP	0.30	0.011	<0.01	0.779

^1^ R^2^ is a statistical parameter to describe the goodness of fit of a regression model.

^2^ Results of relative weight analysis to calculate the relative importance of predictors in the regression model.

## Discussion

The present study investigated the monitoring of low-dose UFH in heparin-spiked blood from healthy individuals and ICU-patients. Sensitivity for ex vivo tested heparin concentrations was demonstrated by ACT, aPTT and anti-Xa. Overall, iSTAT Alinity and Hemochron Signature Elite differed in inter-individual variability and bias. Consistent with Dirkmann et al.,^11^ we found differences in mean-ACT values between iSTAT and Hemochron also for low-dose UFH. In ICU-patients, no relationship between ACT and either UFH dose, aPTT and anti-Xa was observed.

Point-of-care ACT is the most frequently used monitoring tool in heparin anticoagulation in extracorporeal therapies.^12^ Furthermore, manufactures’ investigations reported the sensitivity of ACT for lower heparin concentrations. Here, iSTAT Alinity demonstrates linearity for 0-6 IU/ml heparin (LLOQ: 50 s) and Hemochron for concentrations of 0-2.5 IU/ml using LR-ACT cartridges (LLOQ: 83 s). However, standard ACT target range (180-220 s) according to ELSO recommendation is reached by ex vivo concentrations of 0.3-0.4 IU/ml heparin. In order to perform low-dose heparin monitoring, assays need to be sensitive to heparin < 0.4 IU/ml.

Depending on the primary pathology, coagulation imbalances are induced by inflammation,^13^ disorders in the synthesis of coagulation factors and platelets or metabolic dysfunction.^14^ Especially, extracorporeal circuits initiate a complex thrombo-inflammatory response, involving endothelial, leukocytic and immunological factors.^2^ Therefore, ex vivo analysis of UFH dose and ACT is not transferable to the clinical setting, although it provides useful information about instrumental accuracy and bias.

In principle, laboratory parameters for heparin monitoring include a dependence on biological variables, however, citrated plasma reduces the influence of cellular factors (e.g. hematocrit).^14^ The aPTT Pathromtin^®^ SL assay used for UFH monitoring provides high sensitivity for UFH as well as high sensitivity for lupus anticoagulant. The presence of antiphospholipid antibodies inducing heparin-independent aPTT-prolongation is frequently in critical ill patients.^15^ Therefore, in the ICU-population we performed an aPTT test with a less lupus-anticoagulant-sensitive assay (Dade^®^ Actin^®^ FS), which showed comparable results.

ACT is a whole blood-based measurement technique that has no exclusive sensitivity to UFH and is not capable to differentiate between affecting variables.^16^ However, using iSTAT Alinity and Hemochron Signature Elite, the effect of temperature is excluded, because analyzers perform a standardized sample warm-up to 37°C. Moreover, both instruments feature a protection system to measure a defined sample volume. The influence of viscotic factors including platelet count, fibrinogen level and extreme hematocrit values is predicted for the Hemochron system.^11^ Our results underline the influence of platelets and suggests an influence of antithrombin. In ICU-patients the platelet count, hematocrit and fibrinogen are often outside the normal range. In contrast, the iSTAT Alinity is less affected by the mentioned factors but seems to be influenced by hematocrit and CRP, which are also affected in ICU-patients. Furthermore the system is influenced by coagulation factors, especially thrombin.^11^ Thrombin is integrated in coagulation processes and the inflammatory response and may be present in reduced concentrations depending on heparin-coated extracorporeal systems.^2^ Nevertheless, as we demonstrated, the iSTAT is characterized by a low inter-individual variability and results in a more precise ACT measurement.

Analysis of point-of-care ACT illustrates the absence of approved definition for clotting. The presences of synthetic thrombin splitting products are considered by iSTAT Alinity to indicate expired coagulation, while Hemochron performs a blood flow analysis. Practicing ACT-controlled anticoagulation, manufactures of cardio-respiratory support systems compromise heparin therapy by suggesting distinct ACT-targets for proper operation. Using different ACT methods to reach the identical ACT-target is a risk for critical ill patients to over- or underdose heparin. The establishment of ACT-standards for each detection mechanism in a common effort of manufactures’ and clinical users would be preferable.

Several limitations of our study should be noted. First, for the study in healthy individuals we used ex vivo spiking of samples with heparin. This may not entirely reflect the situation in vivo because heparin binds to cells and is subsequently metabolized, which may affect various fractions of the compound in a different manner and alter its anticoagulant properties. Second, for the ICU-study we cannot exclude certain factors that may influence coagulation results beyond those assessed here. For example, critically ill patients may have unspecific lupus inhibitors that prolong aPTT, which were not checked by the lupus insensitive assay, but would not be expected to affect anti-Xa levels. Furthermore, we did not have an opportunity to assess these patients before starting heparin. This might have helped to address the sensitivity of the different ACT systems to heparin more directly. Moreover this study is of exploratory nature and needs to be confirmed by further studies.

In conclusion, we demonstrated that heparin concentrations of 0-0.4 IU/ml can be accurately measured in healthy individuals after ex vivo spiking with both ACT systems as well as with aPTT or anti-Xa. However, in ICU-patients both ACT systems did not correlate with these low heparin doses and are significantly influenced by different parameters (depending on the device) and may therefore not be suitable for monitoring. Continuative studies are necessary to further analyze these findings.
